# 3D Printing and Virtual Surgical Planning in Oral and Maxillofacial Surgery

**DOI:** 10.3390/jcm11092385

**Published:** 2022-04-24

**Authors:** Adeeb Zoabi, Idan Redenski, Daniel Oren, Adi Kasem, Asaf Zigron, Shadi Daoud, Liad Moskovich, Fares Kablan, Samer Srouji

**Affiliations:** 1 Department of Oral and Maxillofacial Surgery, Galilee College of Dental Sciences, Galilee Medical Center, Nahariya 2210001, Israel; dr.adeebz@gmail.com (A.Z.); idan.redenski@gmail.com (I.R.); dannyoren100@walla.co.il (D.O.); adialkasem@hotmail.com (A.K.); asafzigron@gmail.com (A.Z.); shadi.daoud@mail.huji.ac.il (S.D.); liadmoskovich@gmail.com (L.M.); kablanp1@gmail.com (F.K.); 2 The Azrieli Faculty of Medicine, Bar-Ilan University, Safed 1311502, Israel

**Keywords:** 3D printing, additive manufacturing, waferless, patient specific implants, total join replacement, virtual surgical planning

## Abstract

Compared to traditional manufacturing methods, additive manufacturing and 3D printing stand out in their ability to rapidly fabricate complex structures and precise geometries. The growing need for products with different designs, purposes and materials led to the development of 3D printing, serving as a driving force for the 4th industrial revolution and digitization of manufacturing. 3D printing has had a global impact on healthcare, with patient-customized implants now replacing generic implantable medical devices. This revolution has had a particularly significant impact on oral and maxillofacial surgery, where surgeons rely on precision medicine in everyday practice. Trauma, orthognathic surgery and total joint replacement therapy represent several examples of treatments improved by 3D technologies. The widespread and rapid implementation of 3D technologies in clinical settings has led to the development of point-of-care treatment facilities with in-house infrastructure, enabling surgical teams to participate in the 3D design and manufacturing of devices. 3D technologies have had a tremendous impact on clinical outcomes and on the way clinicians approach treatment planning. The current review offers our perspective on the implementation of 3D-based technologies in the field of oral and maxillofacial surgery, while indicating major clinical applications. Moreover, the current report outlines the 3D printing point-of-care concept in the field of oral and maxillofacial surgery.

## 1. Introduction

### 1.1. Industrial Revolutions and the 3D Printing Era

The first, second and third industrial revolutions, spanning from the 18th century up to 21st century, brought to a major shift in production and manufacturing. Mass-scale manufacturing machines were introduced, alongside significant innovations in communications, electronics and transportation. Process automation emerged and technology moved from analog to digital programming, which significantly impacted computer-monitored production. As a result, the supply of goods underwent dramatic shifts, addressing the demand for increased product volume, variety, design and customization. These culminated with transition towards the 4th industrial revolution during the second decade of the 21st century, and the introduction of additive manufacturing (AM) and 3D printing (3DP) [1].

When compared to traditional manufacturing methods, AM and 3DP, as driving forces of the current revolution [2], stand out in their ability to rapidly fabricate complex structures with complex and highly precise geometries, diverse microarchitectures and hollow spaces or discrete inner objects. Other major advantages include improved design software, marked cost reduction and the simplicity of production, rendering 3D printing accessible to individuals with no previous background in computer aided design (CAD), engineering or additive manufacturing [2,3]. Unlike traditional manufacturing methods, it is based on the fabrication of objects by the sequential addition of material layers [3,4]. The rapidly growing need for products with different designs, purposes and materials has led to the development of various 3D-printing methodologies. As a driving factor of the 4th industrial revolution, 3D printing has had a global impact on healthcare, with 3D-printed, patient-customized therapies replacing outdated methods that rely on systemic, generalized treatment regimes. This 3D-based paradigm shift toward precision medicine has now generated individualized treatment regimens [5]. The AM market in medicine has doubled between 2019 and 2020, reaching 1.65 billion dollars, and is now the third in the industry, second only to the automotive and electronic markets [6]. These changes have had a particularly significant impact on the field of oral and maxillofacial surgery (OMFS), where surgeons rely on precision medicine in everyday practice.

### 1.2. Overview of AM Technologies

Since the first report of 3D printing in 1986 by Charles Hull [7], who used stereolithography (SLA) based on solidifying layers of photopolymer resin, 3D printing technologies have evolved at a staggering pace. AM and 3DP, also known as a form of rapid prototyping, refer to the creation of a physical object from a 3D digital model, typically by laying down or solidifying a material, layer by layer in succession [8].

Among the existing standards for 3D printing terminology, the recently published “ISO/ASTM 52900 Standard Terminology for Additive Manufacturing–General Principles–Terminology” [9] establishes and defines key terms to describe 3DP techniques and infrastructure. We encourage our colleagues to use widely acceptable terms in publications to promote consistency in the literature. This is of upmost importance, specifically in light of the increasing use of 3DP technologies in the field of OMFS [9]. We herein classify AM technologies for construct and implant fabrication into six distinct processes. 

**Figure 1:**
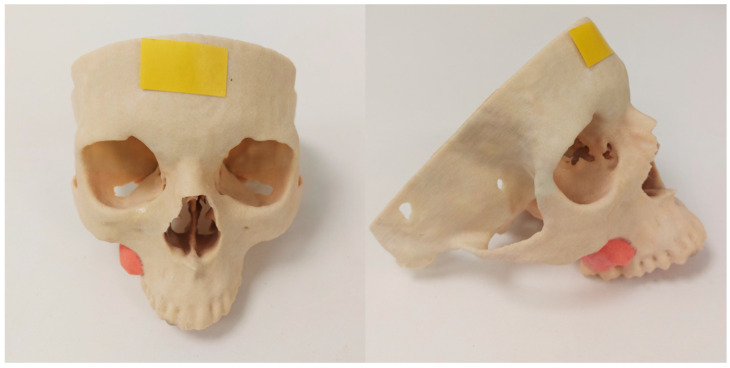
Visualization using 3D printing. 3D printed plaster model fabricated by multi-colored Binder-Jet 3D printing. The lesion outlined in red demonstrates osteosarcoma in the Maxilla.

**Binder jetting** is a process in which liquid solutions are extruded from a printhead and deposited on top of powdered media. Droplets infiltrate the powdered media, resulting in crosslinking of the material, which is followed by introduction of a new layer of material [10,11]. The main advantages of the technology are the low cost of materials and the ability to print in color. However, the low resolution associated with this method and the unset powder and low compressive strength are its major drawbacks. In dentistry and OMFS, this technique is primarily used to create anatomical study models and dentures [12,13,14] (Figure 1).

In **directed energy deposition** (also known as electron beam additive manufacturing [EBAM]), a high-energy electron beam is utilized to selectively melt and fuse a desired metal on a build platform, upon which new material is deposited via a nozzle. These printers offer speed with high temperatures, precluding the need for post-process heat treatment [15]. Moreover, extremely dense products with controlled porosity can be fabricated, such as custom titanium plates and models for cranioplasty [16,17] or mandibular reconstruction [18]. However, the technology, as well as the materials, are costly. Airborne particles are also generated during the fabrication process and may introduce health risks [19]. Printed parts possess a rough surface, and the resolution is low, rendering this technology less popular for accurate medical applications [20].

**Material extrusion** (also known as fused deposition modeling [FDM] and fused filament fabrication [FFF]) is a highly common form of 3D printing, in which a material or polymer is dispensed, in a controlled manner, from a printhead that usually contains a heating apparatus, onto a build platform [21]. The technology offers high-porosity products with variable mechanical strength, depending on the materials used and print settings. Both materials used and printers are low to moderately priced. In clinical settings, sterilization options exist, depending on the printed material [22]. One of the main limitations of the technology stems from the narrow diversity of print materials, which are mainly thermoplastic polymers [23]. Moreover, interlayer bonding is limited [24], and the technology allows for only a low degree of complexity in end-products, making them less than optimal for biomedical applications. Overhangs and support material must be removed manually. Thus, in clinical practice, the technique is mainly used to generate anatomic models and provisional prosthodontics and restorations [25,26].

**Material jetting** (also known as drop on demand [DOD], PolyJet) involves the jetting of a curable medium, such as light-sensitive polymers, onto a build plate via an inkjet printhead [27]. These are cured layer-by-layer while the platform is constantly lowered, with a supportive structure similar to SLA printing. This methodology provides high accuracy and smooth surfaces in a relatively fast and uncostly process. However, the dispensed materials are expensive and messy, and can cause irritation to living tissues [28]. Moreover, heat sterilization is not an option, and products have a limited shelf life. Thus, the main uses in the field are for dental models and provisional prosthodontics [14,29,30].

In **powder bed fusion** (also known as selective laser sintering [SLS] and direct metal printing [DMP]), a powdered medium is dispensed onto a build platform, and then subjected to intense and focused heating, which bonds the powder particles [31]. The materials used are diverse and include elastomers, titanium, composites and metal alloys [32]. The use of lasers makes the process highly accurate, and metal-based products can offer extremely high mechanical integrity [33]. Another major advantage stems from the fact that no support material is required for the fabrication of complex geometries [34]. The end products are autoclavable and can be rapidly produced [35]. The main disadvantage of the process is the heavy infrastructure required for the manufacturing process, as well as the high cost of the technology. The process produces hazardous particle dust, and an elaborate post-production phase may be required, especially due to the rough surface of printed products. This methodology has been applied to produce dental prosthesis, dentures, and implants [36,37].

**Vat polymerization** (also known as stereolithography apparatus [SLA] and direct light processing [DLP]) is a process in which a photosensitive polymer solution within a container or chamber, is cured using a light source [7]. The process is fast, and enables the fabrication of extremely complex constructs with high accuracy. It has proven to be highly accurate in fabricating permanent and temporary restorations, dental models and surgical guides [29,38,39,40,41]. However, the resins used are messy, and for the most part, are not biocompatible and feature poor mechanical properties. End products suffer from a limited shelf life, and may require additional post-processing, as well as rigorous washing steps to avoid extensive release of unpolymerized monomers [42].

**Sheet lamination** (also known as laminated object manufacturing or LOM), relies on the fusion of discrete layers of material to form an object, and is a less popular technique for medical applications.

## 2. 3D Printing in OMFS

### 2.1. The 3DP Point-of-Care Concept

As defined in a recent discussion paper by the FDA [43], a 3D printing point-of-care (3DP PoC) facility, is a physical infrastructure located near or at the treatment site of patients in need of custom-fabricated implants and devices (e.g., hospitals, ambulatory surgical facilities, outpatient treatment facilities, physicians’ offices or dental laboratories). In the last few years, the integration of 3DP PoC laboratories into healthcare facilities has become increasingly more prevalent amongst some of the world’s most highly ranked hospitals and healthcare centers [44]. PoC laboratories are equipped with an infrastructure that usually includes 3D printers, post-processing equipment and appropriate software that enable the digitization of medical images into 3D models. These platforms stand at the core of personalized surgical treatment planning. The establishment of a 3DP PoC facility can bring these technologies closer to the surgeon, making it easier to incorporate them into daily practice, and aiding in achieving optimal clinical outcomes in a diverse set of cases [45,46,47,48]. In 2017, Jacobs and Lin described four distinct usages for 3D printing technologies in the field of craniomaxillofacial surgery [49]. These include contour models, guides, splints, and implants, all of which can be either designed or manufactured at a 3D POC facility.

**Figure 2:**
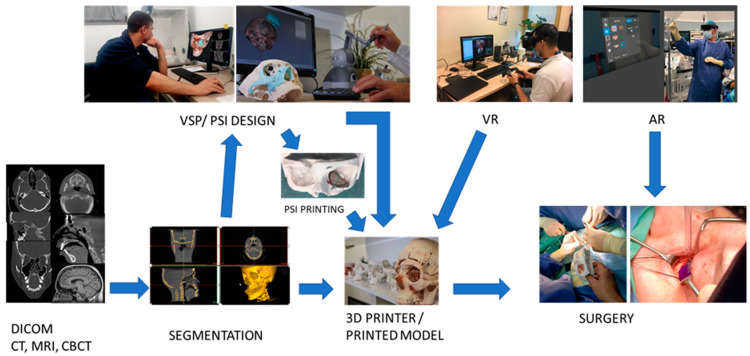
Workflow at the 3DP PoC facility. Patients’ volumetric data obtained after medical imaging is translated to digital imaging and communication in medicine (DICOM) format, followed by segmentation and 3D rendering for virtual surgical planning (VSP) and patient specific implant (PSI) design. Both models and implants are 3D printed, sterilized and subsequently used for surgery. Virtual reality (VR) is used for further evaluation and simulation before surgery. Augmented reality (AR) may assist the surgical team during surgery.

In OMFS departments that utilize 3DP PoC infrastructures, the workflow of each case commences with adequate, high-resolution imaging (Figure 2). Upon admission to the outpatient clinic or the emergency room, patients undergo computed tomography (CT) imaging to visualize the head and neck. Acquisition with a voxel size of more than 1.0 mm [3] may be suboptimal for the purpose of 3D design due to compromised resolution. Proper communication between the radiologists and the digitized treatment planning team at the 3DP PoC center is essential to obtain the imaging needed to enable the accurate design of instruments while avoiding the pitfalls of less-than-optimal anatomical details [50]. Cone beam computed tomography (CBCT) scans and the intraoral scanning of the dental arches may also be warranted, depending on the specific case.

### 2.2. Common 3DP Point-of-Care Workflow in OMFS

**Figure 3:**
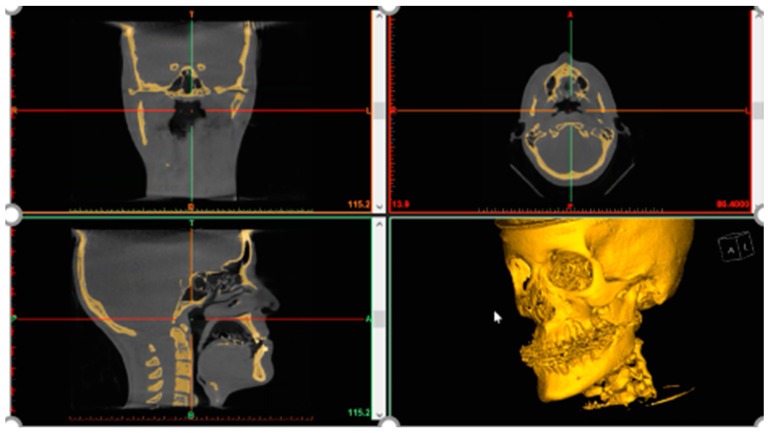
Anatomical segmentation and volumetric data extraction. Mimics 3D evaluation used to delineate threshold bone regions of interest (ROIs).

Following imaging, medical data are obtained in digital imaging and communications in medicine (DICOM) format and segmented using dedicated software, such as D2P (3DSystems) or Mimics In-print (Materialise), both of which are FDA-cleared segmentation and patient data-extraction software. Further segmentation is performed to delineate the region of interest from 2D sections, later to be interpolated into a 3D object (Figure 3). This can be achieved either automatically, manually or in a combined manner, based on image contrast. After additional processing, e.g., noise removal and defect correction, a 3D model in the form of STL data is extracted from patient images, and can be either 3D printed or further designed as a template for guides or patient-specific implants (PSIs), using dedicated design software. These include the FreeForm plus (3DSystems) or the ProPlan CMF (Materialise). Virtual surgical planning (VSP) has had a major impact on the field of OMFS [51,52]. As design software and printing infrastructure become more readily available for surgical teams at 3DP PoC centers, treatment planning that is heavily reliant on individual surgeon expertise is shifting toward a more accurate and comprehensive treatment design process [53]. By incorporating the skills of clinicians on site, as well as together with crosstalk between treating physicians and engineers, the 3DP PoC can tremendously improve surgical outcomes, and provide most of the needs for the surgical team.

The practice of oral and maxillofacial surgery is becoming increasingly demanding and challenging, due to the complex anatomy combined with the growing desire for better, accurate and more aesthetic treatment outcomes. Delicate and precise functions, such as mastication, eye movements, phonetics and facial expression are highly affected by temporomandibular joint (TMJ) pathology, trauma, tooth loss, tumor resection and other pathologies of the face and oral cavity. Treating these conditions requires the new generation of OMF surgeons to become proficient in 3D design and manufacturing for healthcare support [54]. 

## 3. Clinical Applications of 3D Printing in OMFS

The field of OMFS has witnessed significant progress, from wiring through plating, and recently, guided osteotomies and PSIs. Considering the complexity of facial skeleton reconstruction, facial asymmetry repair, orbital volume re-establishment, comminuted fracture reduction and the improvement of aesthetics and functional performance can be extremely challenging. Upon acquisition of the patient’s anatomy using standard medical imaging technologies, a patient-specific treatment plan is designed, following the workflow presented above, i.e., DICOM segmentation, volumetric evaluation and implant design and 3D printing. Anatomic rehabilitation of facial bones can be tackled using a variety of treatment options based on 3D printed models, VSP and digitized fracture reduction and repositioning, virtual osteotomy design, mirroring and surgical guides preparation. These are described in the following section, and are summarized in Table 1.

### 3.1. 3D Planning and Manufacturing for Management of Facial Trauma

The variety of trauma injuries that the OMFS team encounters, as well as the need to reduce the time between pre-operative assessment and treatment, necessitate flexibility and adaptation of both design and AM technologies. The establishment of 3DP PoC facilities within the healthcare campus can reduce the duration of the primary guided reduction and fixation (GRF) process, from hospitalization and up to 3D-based instrument fabrication, down to approximately one week—a reasonable timeframe for the treatment of most OMFS injuries. 

**Figure 4:**
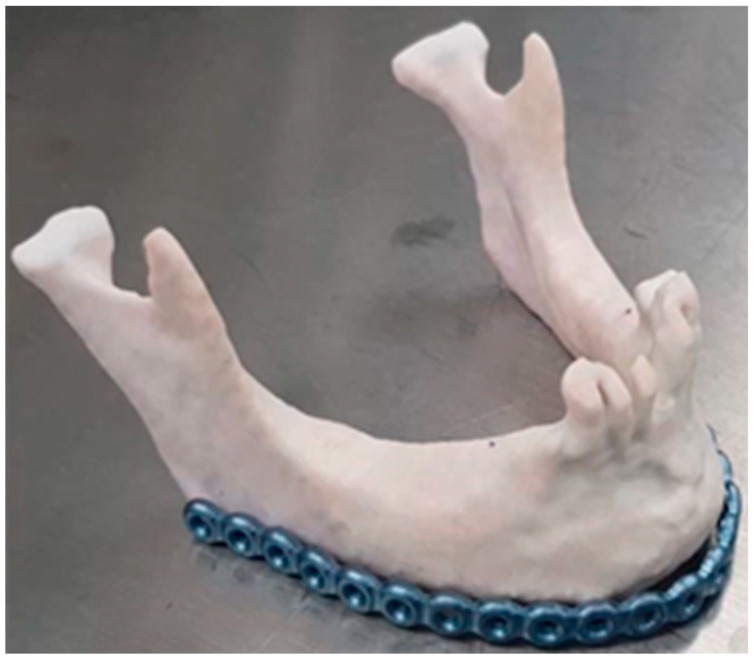
Implants pre-bending on 3D-printed models. Pre-bending of a reconstruction plate for mandibular reconstruction prior to the resection of the symphysial region due to SCC invasion.

Mandibular fracture management commonly involves the printing of the relevant anatomy using desktop FDM, SLA or binder jet 3D printers and pre-bending of fixation plates on models, ref. [55] or the production of custom plating based on VSP [53,64] (Figure 4). In cases of trauma to the anterior mandible and parasymphisial region, fractures can be present both at the site of impact, as well as at one or both condyles, which challenges the accurate reproduction of the inter-condylar distance and occlusion [112]. In these cases, internal fixation planning based on virtual reduction of fractures and patient-specific designs have been shown to minimize postoperative complications, and to enable proper restoration of the intercondylar length [65,66].

**Figure 5:**
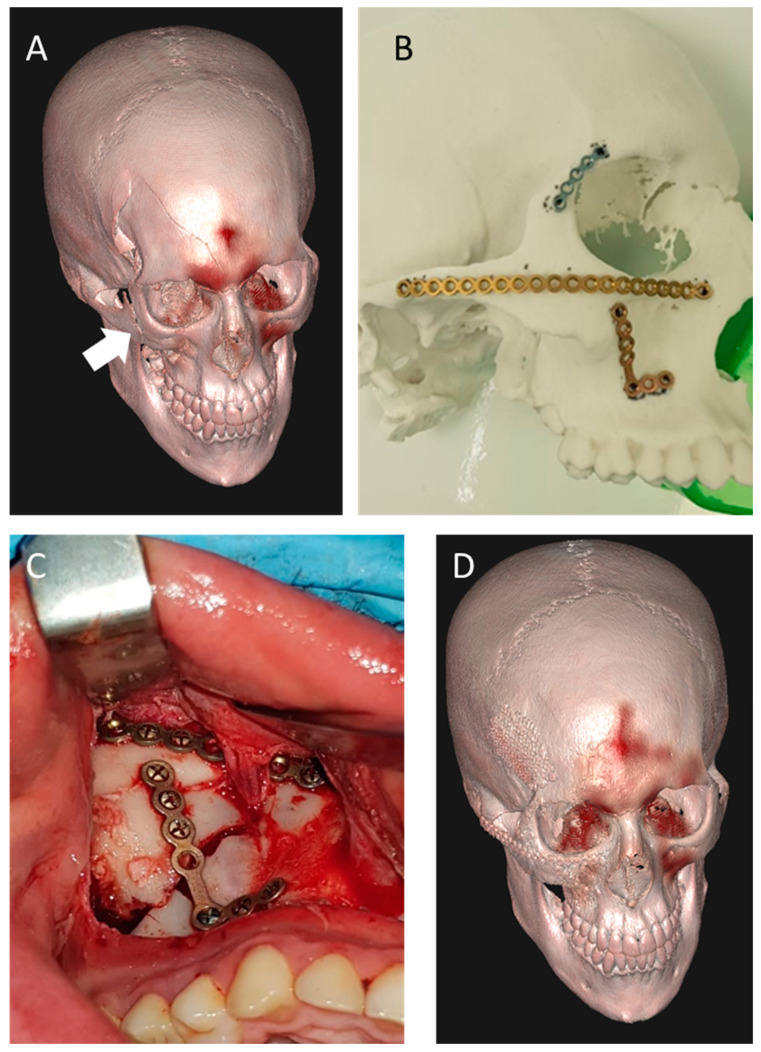
3D design and printing for midface reconstruction. Volumetric representation of a zygomatic complex fracture (white arrow) is obtained, followed by mirroring and pre-bending reconstruction plates based on a 3D printed model (**A**,**B**). Intra-operative installation of pre-bent implants (**C**) and 3D visualization of the postoperative result (**D**).

**Figure 6:**
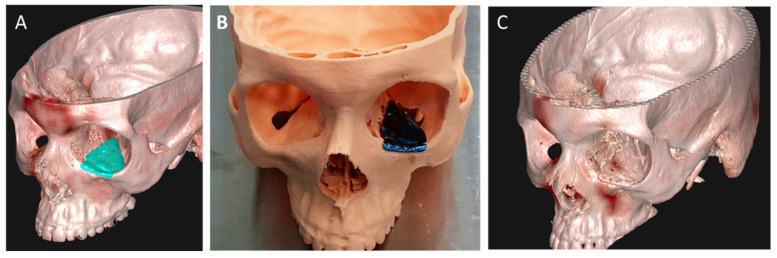
PSI design and 3D printing used for orbital floor reconstruction. VSP-based mirroring is utilized to design PSI for orbital floor reconstruction (**A**). Designed titanium implant fabricated via SLS 3D printing (**B**). Post-operative 3D imaging depicts accuracy of implant adaptation to the damaged anatomy (**C**).

3DP-based treatment of trauma to the midface and zygomatic complex follows a similar workflow, using anatomical models prepared via FDM, SLA or binder jet for the pre-bending of fixation plates [60,61,62] (Figure 5). While pan-facial fractures can be managed by utilizing inter-occlusal relations and fragment repositioning, the occlusion is sometimes insufficient or irrelevant for the reduction of fractures. In these cases, VSP-based design of PSIs for fragment reduction can be extremely beneficial [56,67]. Reconstruction of the orbit follows similar treatment protocols, with preoperative 3D evaluation of the anatomy serving as the new standard of care. Numerous reports on 3D-based treatment approaches describe evolving treatment regimes. Pre-bending of titanium meshes [59,60], bioabsorbable implants [61] and even autologous bone [62] have been reported, using SLA or FDM models of the fractured orbit. Another methodology utilizes mirroring of the intact contralateral anatomy instead of the fractured orbit, which is subsequently 3D printed and used for pre-bending [60,63]. In parallel, the mirrored anatomy can serve as the basis for VSP and subsequent PSI design, usually achieved by utilizing software such as Mimics 3D (Materialise NV Inc., Leuven, Belgium) or FreeForm plus (3DSYSTEMS) and SLS or milling techniques for implant fabrication [68] (Figure 6).

A combination of 3D-based techniques with the numerous avenues available for problem-solving creates a productive setting for unique and inherently unexpected traumatic injuries to the maxillofacial region. Maxillomandibular fixation (MMF) for closed mandibular reduction is an example of a straight-forward treatment regimen that can be simplified using AM technologies. Druelle and colleagues reported on the use of a simple FDM procedure to produce patient-specific rigid arch bars for MMF in a patient suffering from Le fort 1, 2 and 3 fractures [113]. In 2015, Zong et al. reported on the reduction and fixation of a severely fractured condyle in a 14-year-old patient. SLA printing was applied both to visualize the fractured condylar head and to fabricate an anatomical guide for Kirschner wire fixation. Thus, in trauma-related cases, 3DP-based treatment can be highly flexible and clinically beneficial [56,60], and decrease time spent in the operating room [114].

### 3.2. 3D Planning and Manufacturing in Orthognathic Surgery

Pre-operative imaging is a critical stage of patient assessment prior to orthognathic surgery. While conventional 2D radiography is traditionally used for diagnosis, surgical planning and splint fabrication, it bears substantial limitations for orthognathic surgical planning, such as an inherent lack of 3D information on anatomical structures and low-resolution-related inaccuracies, which are carried into the operating theater through suboptimal plaster cast design [115]. These drawbacks have emphasized the importance of both comprehensive recording of patient anatomy and high-resolution imaging during the transfer of the anatomical landmarks onto skeleto-dental models and splints. Optimization of CBCT detail acquisition at lower radiation doses [116] contributed to the implementation of 3D imaging modalities in the field of orthognathic surgery. Pre-operative 3D imaging, particularly CT and CBCT, has become a pillar in the design of treatment plans and in navigation-based guidance of surgeons during procedures [117,118,119]. As a true volumetric technique with a spatial resolution of 100–200 µm voxels, it provides an accurate representation of patient anatomical features [120,121,122,123], which are then transferred into appropriate planning platforms. 

**Figure 7:**
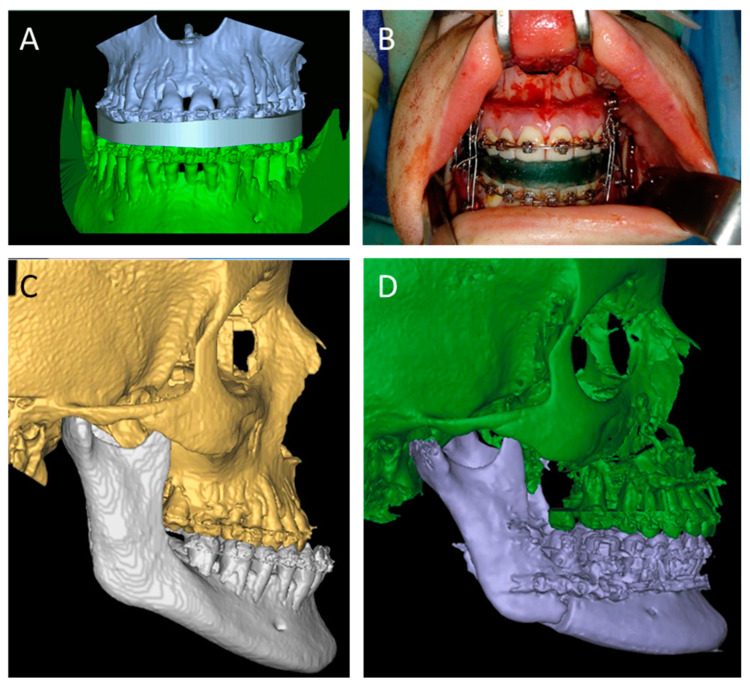
3D printing for orthognathic surgery. VSP for jaw repositioning with virtually created splint in position (**A**) and printer, Intraoperative use of the 3D printed splint via VAT photopolymerization (**B**) Pre-operative (**C**) and post-operative (**D**) CT-based reconstruction.

Traditionally, 2D cephalograms and dental cast models mounted on fully adjustable articulators, and face-bow registration were used for surgical planning [124,125]. In light of the revolutionary 3D techniques and digitization of the pre-surgical process, the dental arches and the skeletal anatomy are not only digitized, but can now also be carefully aligned to yield a composite 3D model of the patient prior to planning. First, considering the low resolution and high rate of artifacts obtained with conventional CT or CBCT [117], the acquisition of a high-resolution scan of the occlusal arches in appropriate relation is key. A major breakthrough was achieved by combination of scanned plaster models, CT scanning of the skeletal anatomy and the use of a reference splint with fiducial markers to create a composite representation of the dento-skeletal system [69,70,71]. CT-based scanning of the dental splint and models, termed a “double CBCT procedure” [72], was also reported, and even a triple CBCT method was described as an “all-in-one” procedure intended to minimize soft tissue deformation during detailed acquisition (Figure 7) [73]. To simplify the process and eliminate the need for fiducial markers, Kim et al. [74] and Noh et al. [75] suggested the use of the iterative closest-point algorithm to super-impose the high-resolution scans of the impression-based dental arches with corresponding craniofacial CT scans. Dental recordings have also seen a major advancement with the introduction of the intra-oral scanner [76], which reliably records occlusal details for composite-model establishment [77,78]. Once segmented data are generated and translated into composite models, they are loaded onto appropriate surgical planning platforms. These include, but are not limited to, Proplan CMF (Materialize, Leuven, Belgium), Maxilim (Medicim NV, Mechelen, Belgium) SimPlant O&O (Materialize, Leuven, Belgium) and Dolphin (Dolphin Imaging and Management Solutions, Chatsworth, CA, USA). 

**Figure 8:**
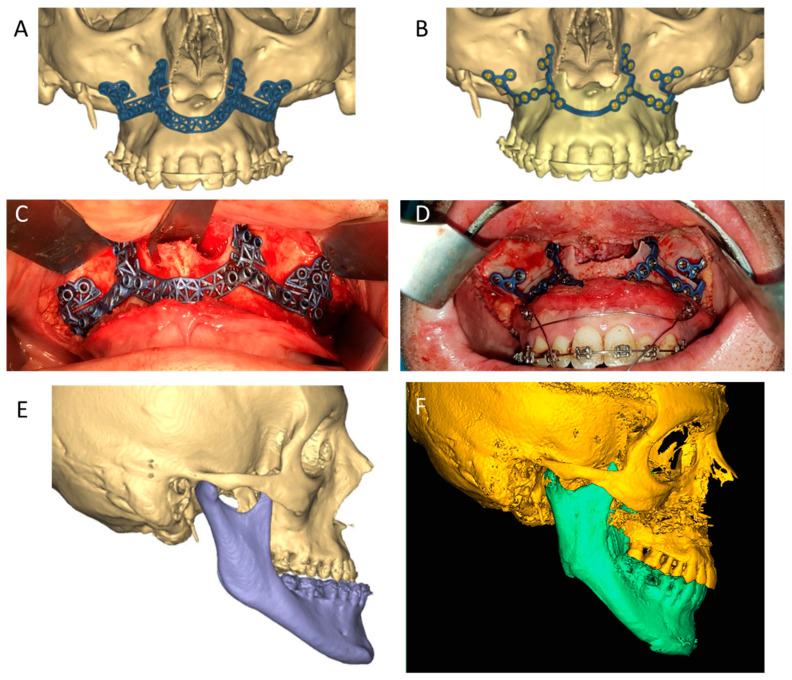
Waferless technique for orthognathic surgery. Design of pre-operative guide and PSI for waferless surgery (**A**,**B**). Intra-operative use of 3D printed titanium guide and PSI (**C**,**D**). Pre- and post-operative 3D reconstruction of the patients’ CS scans (**E**,**F**).

Nowadays, 3D-printed pre-surgical distalizers and power-arm appliances can yield accurate tooth movement in the pre-surgical treatment [126,127], and custom osteotomy guides can be designed to achieve surgical maneuvers that are as close to the 3D planning as possible. 3D printed splints, wafers and guides are used sequentially to position and reproduce the desired occlusal relation, perform the osteotomies and retain both the maxilla and the mandible in their new positions until the designed plating is attached (Figure 7). 3D-printed surgical splints can be fabricated without cutting guides [79,80,81] or incorporated into the design to transfer the exact virtual osteotomies during surgery [82,83,84,85,86,87]. In all cases, and specifically for waferless orthognathic surgery, drill holes of splints are used as reference points for the placement of PSIs to maintain the new jaw position and occlusal relation, eliminating the need for occlusal wafers and simplifying the surgical process (Figure 8). Studies evaluating traditional versus 3D-based planning and execution of orthognathic surgery indicated higher regression of the end result when 2D analysis was used, both in the horizontal and vertical dimensions [128,129]. Shaheen et al. reported on highly accurate surgical outcomes for procedures planned using VSP [130], and Bengtsson et al. reported higher accuracy in 3D-based orthognathic surgeries as opposed to 2D-based surgeries in predicting the maxillary outcome [129]. As follow-ups continue and the use of VSP for orthognathic surgery becomes increasingly popular amongst clinicians in the field, more reports comparing 2D and 3D treatment are expected to emerge and shed light on the additional advantages of 3D printing in orthognathic surgery.

### 3.3. 3D-Based Digitization and Planning for Maxillofacial Tumor Resection and Reconstruction

Oral malignancies account for 3% of all cancer cases diagnosed annually worldwide [131]. Unfortunately, about half of the oral cavity cancers are detected at an advanced stage, which leads to poor prognosis, with high complication and mortality rates [132]. 3D design, 3DP technologies and VSP and the shift from surgeon-dependent resection toward precise, 3D-based evaluation of tumor and surgical margins [133,134] have made a marked impact on the field of tumor ablation and control of surgical margins [135].

Major maxillofacial tumor resection commences with segmentation based on CT scans, to delineate the cancerous lesion and non-compromised healthy tissues. The relevant anatomy is translated to the DICOM format, allowing the surgeon to create virtual 3D models of the target regions and simulate the surgical beds and donor sites as necessary [88]. The readers are referred to the review of available software for VSP and resection design, recently published by Gustaaf et al. [136]. Cutting guides considering the acceptable resection margins are designed to accurately transfer positions and angulations of the osteotomies to the operating team [45,90], and 3D models are also useful to assess the risk for post-operative fractures following tumor resection [89].

**Figure 9:**
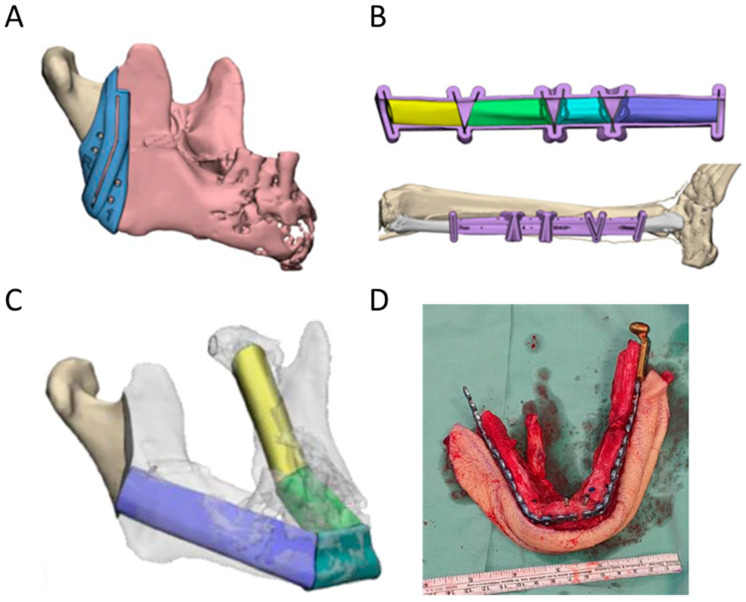
3D design and printing for mandibular reconstruction using the fibula-free flap. osteotomy guides for both the cancerous lesion in the mandible (**A**) and fibular tissue harvest (**B**) were designed based on the patient’s anatomy. 3D VSP-based reconstruction of the mandible and subsequent pre-bent reconstruction plate with harvested fibular flap (**C**,**D**) VSP images courtesy of 3D4OP.

In cases of severe mandibular resection, the free fibular flap is indicated for reconstruction [137] (Figure 9). Virtually designed osteotomies guide the surgeons during autologous graft harvest, enable accurate graft fit, and reduce surgical time in the reconstruction of the maxilla or the mandible [91]. Fixation techniques after tumor-related reconstructions have also witnessed tremendous progress, from pre-bent reconstruction plating [92,93,94] to VSP-based PSIs. While the majority of PSIs are fabricated via SLS 3D printing of titanium [90,95], milled or filament-fabricated PEEK PSIs are emerging as additional options for maxillofacial reconstruction in light of their proven use in orthopedic surgery [96] and resilience to stress and adaptable geometry [97,98], with better restoration of the original anatomy as compared to titanium plating alone [99]. Several preliminary studies have compared the biological and mechanical features of titanium implants with alternatives such as PEEK. Initial findings indicated that PEEK alone is inferior to titanium in withstanding the cyclic and displacement forces applied and requires reinforcement [138]. Additional reports comparing materials for PSI production and their long-term biological integration will shed more light on other alternatives.

### 3.4. Total Joint Replacement (TJR) in the Era of 3D Printing

TMJ disorders can result from intra- or extraarticular pathologies, manifesting as pain, limited mouth opening, malocclusion and jaw deformity [139,140]. In the case of end-stage TMJ disorders, severe degenerative joint disease is not responsive to conservative therapy and necessitates surgical intervention, since the joint components cannot be salvaged [141]. In these cases, reconstruction of the TMJ is performed to restore both function and alleviate symptoms [104,105,142]. Reconstruction of the TMJ has evolved in the past century, and has integrated various materials, with mixed results [143,144,145,146]. As TMJ reconstruction surgeries became more common, implant design, materials used and surgical techniques rapidly evolved, and CAD/CAM was implemented for the fabrication of stock or custom TMJ replacements [100,104,106].

Although most joint components were traditionally made by CNC milling, newer generations of joint constructs are manufactured by the 3D printing of metals, and employ metal AM techniques such as SLM, DMLS and EBAM [147]. Common implant systems for TJR have been extensively reviewed by Guarda-Nardini [148], and also recently by Mehorotra et al. [149]. The stock system consists of three universally sized mandibular condyle and fossa components [150], while patient-matched and fully customized options also exist. For patient-matched implants, both the fossa and the condyle have a universal design template, which is digitally sized preoperatively according to the patient’s CT scans. When the patient’s anatomy does not fit the template, the fully customized route is taken, and a prosthesis is designed to accommodate the extreme variance [104].

**Figure 10:**
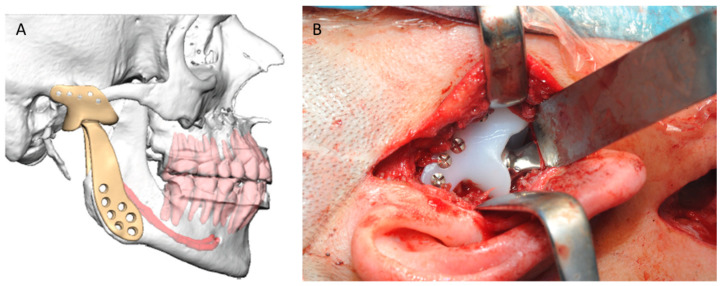
3D-based TJR. VSP (**A**) and intraoperative placement of the patient-specific implant to the mandible and fossa (**B**).

Some of the advantages of PSIs over stock implants derive from the customization of the implant and its components: size, shape, screw length and position, as well as the material itself in case of allergy to Co-Cr-Mo metal alloys. Likewise, surgical guides for precise bone removal and the minimization of risk of nerve damage by fixation screws placement are also available [100,101,102,103] (Figure 10).

The major disadvantages of customized systems are their high costs and meticulous design process [107,108,109]. Still, the potential to reduce surgical time, bone resections, post-operative hospitalization and complications using custom systems has to be considered in end-stage joints disorders. Only two patient-specific (PS) TJR systems are currently approved by the US Food and Drug Administration (FDA): Zimmer-Biomet Inc. (Jacksonville, FL, USA), which markets stock and PS TMJ implants and TMJ Concepts Inc. (Ventura, CA, USA), which specializes in custom TMJ implants [100,105,106,110]. While reports comparing the outcomes of custom vs. PS TJR systems are still limited, results indicate acceptable success rates and surgical outcomes using PS TJR implants [104,111].

### 3.5. Virtual Reality (VR) and Augmented Reality (AR) for OMFS

**Figure 11:**
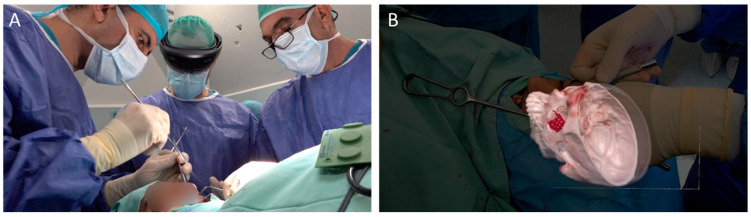
Augmented reality for orbital reconstruction. A surgeon is wearing the head-mounted glasses (**A**). Visualization and projection of 3D data within the operative field, depicting co-registration of the PSI onto the orbital fracture (**B**).

As the OMFS field is consistently engaged by 3D printing and VSP, further adaptations are mandatory to promote both an understanding of and proficiency in 3D-based technologies. Virtual reality (VR) and augmented reality (AR) will be key tools in achieving these goals. In simple terms, VR is an immersive experience where physical objects and environments are replaced by digitized ones. Hand-held controllers and devices with haptic feedback are used to interact with the virtual surgical environment, and the physical spatial position of the controllers is tracked and applied to the simulated surgical instrument [151]. VR is extensively used for pre-operative anatomical assessment, VSP and intraoperative navigation [152,153,154,155]. AR, in contrast to conventional navigation, image-guided systems and VR, enables the operator to co-register digital models and data directly onto the surgical bed. This projection onto the real world enhances the physical experience, while eliminating the need to look away from the patient [156] (Figure 11). Current state-of-the-art technology utilizes several technologies, such as an optical see-through (OST) display that enables augmented data to overlay the physical world viewed by the operator. This is done using devices such as the Google Glass or HoloLens. Such projection of digital-to-real anatomy has already been applied in neurosurgical procedures [157], as well as in orthognathic surgery [158,159], mandibular reconstruction [160] and facial deformity repair procedures [161,162,163]. Thus far, AR-enhanced surgeries have reached high levels of accuracy [164], while avoiding damage to critical anatomical structures [165]. In an educational context, AR can aid in the training of medical students and surgeons, since the spatial complexity of internal compartments can be easily visualized [166,167]. Once established as safe and accurate, AR promises to bear a tremendous impact on medicine in general, and on maxillofacial surgery in particular.

### 3.6. 3D-Based Tissue Engineering and Translational Medicine in OMFS

Tissue engineering (TE) is an interdisciplinary field set to combine concepts of life sciences and engineering to develop biological substitutes for failing tissues and organs [168]. Ever-advancing technologies enabled the research and production of complex tissue engineered constructs (TECs) that combine cellular components with biocompatible three-dimensional biomaterials, which can enhance cellular growth, attachment and differentiation. The basic process of TEC fabrication includes the isolation of cells from a patient, in-vitro expansion and differentiation of cell populations and cellular loading onto a three-dimensional construct. These constructs are further incubated to allow further architectural organization and biological maturation, until they can be considered for transplantation [169,170]. In the field of OMFS, the TE revolution will have a significant impact. Bone TE constructs (TECs) cultured with appropriate stem cells harvested and expanded from patients will soon take their place in the clinically-oriented reconstruction of the facial skeleton [171,172]. As for cartilaginous TECs, the regeneration of cartilage in general and of the mechanically complex TMJ disc, in particular, is a fundamental goal, with considerable challenges to overcome [173,174].

As both the field of TE and 3DP technologies are evolving, personalized laboratory fabricated off-the-shelf TECs are already under development. Combining the biomimetic nature of autologous substances and cells with the rapid and complex 3DP of implants, the need for bone tissue harvest could soon be a thing of the past. With 3D design and fabrication methods rapidly introduced into TE, biologically inspired 3D bone constructs are being experimented in preclinical and translational studies. One breakthrough reported by Bhumiratana et al. [175], and later by Chen et al. [176], involved 3D bones accurately milled and implanted in large animal models, which led to the rehabilitation of a large portion of the ramus and the condyle. The use of 3DP for bone TE with materials such as polycaprolactone (PCL) and calcium-phosphate cements (CPCs) has also been reported, with FDM printers fabricating human-scaled structures for craniofacial rehabilitation [177,178]. 3D bioprinting, a form of 3DP, focuses on the organized deposition of biological substances (bioinks) and cells [179,180] with several key advantages over 3DP of non-biological substances. These include the ability to directly incorporate cells during the printing process [181,182], 3DP of discrete biological compartments using support baths [183,184] and the implementation of in-situ 3DP of constructs [185,186]. The ability to bioprint craniofacial structures is currently the subject of exciting preclinical studies [187,188]. However, structural durability and size of bioprinted constructs are issues that remain to be overcome before they can be applied in clinical settings.

## 4. Discussion, Challenges and Future Prospects

The use of 3D technology and virtual planning for medical interventions, ranging from simple surgical procedures [189] and up to fracture reduction and defect repair [190], has brought to a marked improvement in clinical outcomes. Mirroring of the unaffected side, followed by printing of models, pre-bending of commercial plates or meshes or PSI design, have enhanced accuracy and proven time-efficient [55,190,191,192]. Ballard et al. reported that the application of 3D technologies in OMFS can save up to an average of 83 min per surgery when pre-designed surgical guides are used, and more than 60 dollars per minute of surgery [55]. In orthognathic surgery, VSP can enhance the surgeon’s comprehension of the patient-specific anatomy, and enable a computerized workflow for repositioning of the jaws, rendering previous 2D-based methodologies obsolete. Moreover, 3D-planned treatment regimens have been shown to enhance accuracy and outcomes [87,115,193]. In oncology-related reconstructive surgery necessitating both a neck dissection and free microvascular flaps, time is of essence. Surgical preparation with 3D-based harvest guides and VSP-based reconstructive guides dramatically improve the surgical outcome [50,194,195].

Some limitations to the use of 3D printed implants still need to be tackled. Metallic residues and surface topology may elicit an unfavorable response [196] emphasizing the importance of the post-processing of implants [197]. Since sterilization of printed metal or polymeric implants remains an issue, materials used for PSI manufacturing are also expected to undergo tremendous advances by combining antimicrobial substances and therapeutic agents, as has been recently described in other dental and surgical fields [198,199,200,201]. Moreover, the development of 3D technologies will continue to enhance the control over microarchitecture, porosity, stress-shielding and load-bearing of implants, allowing better osteointegration [202,203,204]. Advancement from biocompatible metals toward bioactive, drug-releasing resorbable implants marks one of the upcoming surgical revolutions. The lack of regulation regarding 3DP PSIs is pushing authorities worldwide to oversee the implementation of these technologies in clinical settings and other fields [205,206]. The reader is referred to a recent comprehensive review on the matter by Gupta et al. [207].

Conventional oral and maxillofacial surgery practices are being continuously challenged by the increasing demand for improved and more accurate treatment outcomes. Delicate and meticulous functions such as mastication, eye movement, phonetics and facial expression are all highly affected by maxillofacial pathology and trauma. Treating these conditions requires proficiency and training with design platforms, different implant materials and AM systems [54]. The establishment of 3D PoC facilities can bring these technologies closer to the surgeon, thereby making them easier to incorporate into daily practice and improving clinical outcomes [45,46,47,48].

## 5. Conclusions

The implementation of 3D technologies in implant design and manufacture is ushering a new revolution into the OMFS field. The advantages of the 3D-based revolution in OMFS are obvious and well-established: efficiency, accuracy and reaching an optimal clinical outcome. While their main drawbacks are the high cost and the need for additional training and heavy infrastructure, these obstacles can be overcome by establishing 3D PoC centers within healthcare facilities. In light of the marked impact these technologies are having on the field, it is our opinion that we, as clinicians, actively promote and implement them in our everyday work regime, in order to further expand the boundaries of the field and bring it closer to meeting its full potential.

## Figures and Tables

**Table 1:** Summary of major 3D applications in oral and maxillofacial surgery. Major fields in OMFS impacted by 3D printing technologies. VSP—virtual surgical planning. CBCT—cone beam computed tomography. PSI—patient specific implants. TJR—total joint replacment.

	Application	References
3D planning and manufacturing for management of facial trauma	pre-bending of fixation plates on anatomical models	Mandible—[55] Midface—[56,57,58] Orbit—[59,60,61,62,63]
	production of custom plating based on VSP	Mandible—[53,64,65,66] Midface—[56,67] Orbit—[68]
3D Planning and Manufacturing in Orthognathic Surgery	Composite models based on fiducial markers	[69,70,71]
	Composite models based on repeated CBCT scans and data obtained using oral scanners	[72,73,74,75,76,77,78]
	VSP-based splints and cutting guides	[79,80,81,82,83,84]
	VSP design for Splintless\waferless surgery	[80,85,86,87]
3D-Based Digitization and Planning for Maxillofacial Tumor Resection and Reconstruction	3D study models of resection sites	[88,89]
	Osteotomy guides	[45,90,91]
	Pre-bending of Reconstruction plates	[92,93,94]
	VSP-based PSIs	[90,95,96,97,98,99]
Total Joint Replacement (TJR) in the Era of 3D Printing	Design of cutting guides for TJR	[100,101,102,103]
	Custom and VSP-based TJR implants	[100,104,105,106,107,108,109,110,111]

## Data Availability

Not applicable.
